# Development of a receptor based signal amplified fluorescence polarization assay for multi-detection of 35 sulfonamides in pork

**DOI:** 10.1016/j.fochx.2023.100867

**Published:** 2023-09-06

**Authors:** Tong He, Peng Lei Cui, Shuai Zhang, Yu Hang Fan, Qiu Shi Jin, Jian Ping Wang

**Affiliations:** a College of Veterinary Medicine, Hebei Agricultural University, Baoding, Hebei 071000, China; b College of Science, Hebei Agricultural University, Baoding, Hebei 071000, China

**Keywords:** Sulfonamides, Dihydropteroate synthase of *Staphylococcus aureus*, Mutant, Signal-amplified fluorescence polarization assay, Pork

## Abstract

With the increasing focus on food security, a screening method with high-throughput, ultra-sensitivity, and user-friendly operation is urgently needed for monitoring of sulfonamides residues in animal-derived foods. In this study, the sulfonamides’ receptor dihydropteroate synthase of *Staphylococcus aureus* was subjected to saturate mutation, and a mutant with higher affinities for sulfonamides was obtained. The mutant was then used as recognition material to establish a fluorescence polarization assay for determination of 35 sulfonamides in pork. Due to the use of an enhanced fluorescent tracer containing two fluorophore molecules, the sensitivities for the 35 sulfonamides were improved for 2.8–8.6 folds (LODs 0.03–1.16 ng/mL) in comparison with using conventional fluorescent tracer. The present method outperformed all previous fluorescence polarization (immuno)assays for sulfonamides due to its broader spectrum, higher sensitivity, and shorter assay time. Furthermore, this is the first study reporting an enhanced fluorescence polarization assay for determination of small molecule substance.

## Highlights

• A SaDHPS is first mutated by using the multipoint mutagenesis technique.
• The recognition mechanisms of mutant for 35 sulfonamides are first studied.
• A signal-amplified FPA is first developed for the detection of the 35 SAs.
• The method’s performances are generally better than the previous FPIA.

## 1 Introduction

Sulfonamides (SAs), as the first class of synthetic antibiotics, are widely used for the treatment of different infections caused by bacteria in human being, livestock, and poultry due to their broad-spectrum antimicrobial properties (Bertacine Dias, Santos, Libreros-Zúñiga, Ribeiro, & Chavez-Pacheco, 2018). However, the overuse of SAs in domestic animal will unavoidably lead to their residues in edible animal tissues, and these residues can cause different health hazards to the consumers, such as allergic reaction, dysbacteriosis, suppression of enzyme activity, alteration of intestinal microflora, and promotion of sustainable form of pathogens (Ovung and Bhattacharyya, 2021, Duan et al., 2022). Furthermore, some SAs have been proved to show hemotoxicity and carcinogenic effect, e.g. sulfamethazine. Therefore, the monitoring SAs residue in animal-derived foods is crucial.

Among the reported analytical methods for SAs, immunoassay is most frequently used for high-throughput analysis of large-scale samples because of its short assay time, simple operation, and low cost. Up to now, many immunoassays, such as enzyme linked immunosorbent assay, fluorescence polarization immunoassay, immunochromatographic strip and immunosensor, have been reported for the detection of SAs (Zhang, Liu, & Wang, 2017). However, the detection performances of these immunoassays are various, which is because the recognition abilities of the used antibodies are various. In other words, a single specific antibody is far from meeting the practical needs. By now, several antibodies with group specificity for SAs have been reported (Chen et al., 2017, Chen et al., 2017, Li et al., 2019, Li et al., 2020, Wang et al., 2013), but the broadest recognition spectrum is limited to recognize 32 SAs with uneven affinities, i.e. not capable of recognizing all SAs. Moreover, the preparation of an antibody requires many laboratory animals, and the process is high cost, time-consuming, and labor-intensive. Therefore, there is an urgent need to find alternative reagent to displace antibody for development of immunoassay.

In comparison with antibody, receptor has several obvious advantages. First, a receptor is usual the target of one class of drugs, so it can recognize all its ligands. Second, a receptor can be obtained by using simple expression technique, i.e. the use of laboratory animal is avoided. Third, the expression of a receptor needs only several days. Fourth, the cost is low. Fifth, the affinity of a receptor for its ligand can be improved by simple directional evolution, which is simpler and more convenient than the evolution of an antibody. For example, a mutant of penicillin binding protein is obtained by site-directed mutagenesis, and the obtained mutant protein shows higher affinity to 33 β-lactam drugs than the parental protein (Ning et al., 2019). In our recent study, a Tet repressor protein is evolved by using single-site mutation technique, and the resulting mutant shows significantly increased affinities for tetracycline drugs with the sensitivity improved for up to 13 folds (Wang, Xia, Liu, Wang, & Liu, 2019). Therefore, the receptor-based multi-screening method has a promising prospect. As the results, a variety of receptors have been used as recognition reagents to develop pseudo immunoassays for the detection of veterinary drugs (Ahmeda et al., 2017).

Dihydropteroate synthase (DHPS) is an enzyme in bacteria that can catalyze the synthesis of the folate intermediate 7,8-dihydropteroate based on *para*-aminobenzoic acid (PABA) (Babaoglu, Qi, Lee, & White, 2004). SAs can compete with PABA and bind with DHPS to disrupt the folate pathway due to their similar structure (Brown, 1962, Yun et al., 2012). So, DHPS is the receptor of SAs that should recognize all SAs species. By now, there have been several DHPS-based pseudo-ELISA methods (He et al., 2022, Liang et al., 2020, Liang et al., 2019, Liang et al., 2013) and fluorescence polarization assays (He et al., 2022, Wang et al., 2015) reported for the detection of SAs residues. Among these analytical methods, fluorescence polarization assay is the simplest, rapidest, and most suitable tool for high-throughput analysis of large-scale samples because it does not require the conventional blocking, incubating, and washing steps. In our recent study, the DHPS of *Staphylococcus aureus* (SaDHPS) is obtained that can recognize 31 SAs, and the SaDHPS based FPA can be used for screening of the 31 SAs with the IC_50_ in the range of 14.1–89.5 ng/mL (He, Liu, & Wang, 2021). However, all of the used fluorescent tracers in the previous fluorescence polarization (immuno)assays contain only one fluorophore molecule, so the method sensitivities are fully dependent on the fluorescent property of the used fluorophore. As the results, many researchers have tried to synthesize the novel and sophisticated fluorophores with high fluorescent quantum yield with the aim of increasing the sensitivity of a fluorescence method (Lavism, 2017).

For improving the method sensitivity, the use of signal amplification strategy is an effective and promising pathway (He et al., 2022, He et al., 2021, He et al., 2021, Cai et al., 2015, Qiao and Cai, 2021). In our previous study, a signal-amplified pseudo-ELISA is developed based on the streptavidinated-biotinylated horseradish peroxidase system, which contains more horseradish peroxidase molecules, so the method sensitivities are improved for 32–88 folds (He, Cui, Liu, Feng, & Wang, 2022). In theory, therefore, a fluorescent tracer containing more fluorophore molecules should enhance the fluorescence signal and consequently increase the sensitivity of a fluorescent method. As far as we know, however, an FPA method based on such signal-amplified fluorescent tracer has not yet been reported.

For improving the recognition ability, our recently produced SaDHPS was evolved by using multipoint mutagenesis technique to produce a mutant, and its intermolecular interaction mechanisms with 35 SAs were investigated. Meanwhile, we synthesized a conventional fluorescent tracer containing one fluorophore molecule and a novel fluorescent tracer containing two fluorophore molecules, with which to verify if such signal amplification strategy can improve the sensitivity of a fluorescent method. Finally, the above reagents were combined to develop a FPA for multi-screening of the 35 SAs in pork.

## 2 Materials and methods

### 2.1 Reagents

The standards of 28 SAs were obtained from Dr. Ehrenstorfer (Ausburg, Germany): sulfanilamide (SN), sulfamethizole (SMT), sulfathiazole (STZ), sulfacetamide (SA), sulfaethoxypyridazine (SEP), sulfaclozine (SCZ), sulfadiazine (SD), sulfamethoxypyridazine (SMP), sulfaquinoxaline (SQ), sulfabenzamide (SB), sulfaguanidine (SG), sulfamethazine (SM2), sulfamerazine (SMR), salfalene (SL), sulfamonomethoxine (SMM), sulfamethoxydiazine (SMD), sulfamoxole (SXL), sulfamethoxazole (SMZ), sulfisomidine (SIM), sulfamethoxypyrimidine (SPD), sulfaphenazole (SPA), sulfisoxazole (SIZ),phthalylsulfathiazole (PST), sulfabenz (SZ), sulfadoxine (SDX), sulfachloropyridazine (SCP), sulfasalazine (SSZ) and sulfapyridine (SPY). The standards of 4 SAs were purchased from Shanghai Yuanye Biological Technology Co., Ltd (Shanghai, China): *N*-acetylsulfadiazine (ASZ), acetyl sulfisoxazole (AS), benzenesulfonamide (BSN), 2-methylbenzene-1-sulfonamide (MBS). The standards of 2 SAs (*N*-acetyl sulfamethoxazole (ASM) and sulfasuccinamide (SSA)) were from Aladdin Biochemical Technology Co., Ltd (Shanghai, China). The standards of 3,5-dichlorosulfanilamide (DCS), fluorescein isothiocyanate (FITC), 4-nitrobenzoyl chloride, 3,5-dinitrobenzoyl chlorideand palladium on carbon were obtained from Macklin Biochemical Co., Ltd (Shanghai, China).

The remaining chemical reagents at analytical grade were from Shanghai Saen Chemical Company (Shanghai, China). Dihydropteridine pyrophosphate (DHPPP, the first substrate of DHPS) was synthesized according to previous research (Wang, Liang, Wen, Zhang, Li, & Shen, 2015). Black 96-well microplates were purchased from Jingan Biotechnology Co., Ltd. (Shanghai, China). The SDS-PAGE gel preparation kit was from Beijing ComWin Biotech Co., Ltd. (Beijing, China). The primers were synthesized at Beijing Liuhe Huada Gene Technology Company (Beijing, China).

The standard stock solutions of the 35 SAs were prepared with methanol (10.0 μg/mL), and their working solutions at a series of concentrations (0.1–500 ng/mL) were prepared with deionized water. The working solution of fluorescent tracer was prepared with deionized water. Phosphate-buffered saline (PBS) was obtained by dissolving 1.15 g of Na_2_HPO_4_, 0.2 g of KH_2_PO_4_, 0.2 g of KCl, and 8.0 g of NaCl in 1000 mL of water. The reaction buffer (pH = 8.0) was PBS containing 4 mM of DHPPP and 40 mM of Mg^2+^.

### 2.2 Virtual mutation

In the complexes of SaDHPS with the 35 SAs, the amino acids within 5 Å of the SAs molecules were exposed to saturate virtual multipoint mutation by utilizing YASARA 16.2.18 (Biosciences GmbH, Austria). If the substitution of some amino acids with others could make the protein more stable or show higher binding energies for most of the SAs, then these amino acids were selected as the mutagenesis sites. During the experiments, it was found that Arg204 and Pro216 were the optimum mutagenesis sites, and the Arg204 was mutated to His and the Pro216 was mutated to Arg to produce the SaDHPS mutant.

### 2.3 Molecular docking

The molecular docking experiments between the SaDHPS mutant and 35 SAs were performed as follows. The 3D conformation of the parental SaDHPS (PDB ID: 1AD1) was imported into the YASARA software for amino acid mutation and followed by energy minimization. During the molecular docking, the mutant was first docked with DHPPP (the first substrate for DHPS) to obtain the mutant-DHPPP complex. After that, the binding pocket was located by docking PABA with the mutant-DHPPP complex. The 35 SAs were then docked into the binding pocket separately to study the intermolecular forces, binding energies, contact amino acids, and binding sites.

### 2.4 Production of SaDHPS mutant

The mutant gene was inserted into an express vector (pET-32a) by Sangon Biotech Co., Ltd (Shanghai, China), and the resulting recombinant plasmids were characterized by using polymerase chain reaction (PCR) (primers: forward, ATGACTAAAACAAAAATTATGGGCATATTAAAC; reverse, TTAAGAAAAATTGTGTCTTGCATTTTCA) and double digestion (*Bam*HI and *Xho*I). The positive recombinant plasmids were transformed into the *E. coli* competent cell (BL21 D3) to express the mutant, and the subsequent purification was performed by using a Ni-agarose resin column as the mutant was fused with HIS-tag. After that, the pure mutant was analyzed by SDS-PAGE electrophoresis and characterized by western blotting analysis. The detailed expression, purification and characterization procedures were shown in our recent report (He, Liu, & Wang, 2021).

### 2.5 Synthesis of enhanced fluorescent tracer

The enhanced fluorescent tracer containing two fluorophore molecules was synthesized as the route shown in Fig. 2A. The first step was to couple SIZ with dinitrobenzoyl chloride to form an intermediate, and the next was to couple fluorescein isothiocyanate (FITC) with the intermediate to prepare SIZ-2FITC. For comparison, SIZ-FITC, which contains one fluorophore molecule, was also synthesized using the comparable steps: SIZ was first coupled with nitrobenzoyl chloride, and the resulting intermediate was then coupled with FITC (Fig. 2A).

The synthesis procedures of two fluorescent tracers were as follows. Briefly, 2 mmol of SIZ, 2 mmol of 4-nitrobenzoyl chloride (or 3, 5-dinitrobenzoyl chloride), and 2 mL of triethylamine were added in 20 mL of tetrahydrofuran to react overnight at room temperature. Upon completion, the mixture was filtered and the solution was dried in a rotary evaporator at 40 ℃ to obtain intermediate 1 (or intermediate 3). Then intermediate 1 (or intermediate 3) was dissolved in 25 mL of absolute ethanol, then 10 mg of Palladium on Carbon (Pd/C) and an appropriate volume of hydrazine hydrate (more than 10 equivalents of –NO_2_) were added to be stirred under the protection of nitrogen stream. After that, the mixture was refluxed at 80 ℃ for 72 h. Upon completion, the solvent was evaporated in a rotary evaporator at 65 ℃ and the resulting solid powder was washed with 10 mL of absolute ethanol. After filtration, a solid mixture containing intermediate 2 (intermediate 4), Pd/c, and impurities was obtained. To obtain purified intermediate 2 (intermediate 4), the above solid mixture was dissolved in 10 mL of N, *N*-dimethylformamide (DMF) and filtered with a 0.45 μm nylon filter membrane. The filtrate was then mixed with the proper volume of deionized water (approximately 30 times the DMF) to precipitate the product. Upon completion, the supernatant was removed after centrifugation at 5,000 rpm for 5 min. The sediment was washed and vacuum dried to obtain the intermediate 2 (C_18_H_18_N_4_O_4_S^+^, HRMS (*m*/*z*) 386.13; Fig. S1A) and the intermediate 4 (C_18_H_19_N_5_O_4_S^+^, HRMS (*m*/*z*) 402.12; Fig. S1B).

Subsequently, 0.01 mmol of intermediate 2, 0.01 mmol of FITC (or 0.01 mmol of intermediate 4, 0.02 mmol of FITC), and 50 μL of triethylamine were mixed in 5.0 mL of methanol. After reacting overnight at room temperature, the reactant was separated by a thin-layer chromatography technique, and the target product (Rf_SIZ-FITC_: 0.21; Rf_SIZ-2FITC_: 0.16) was scraped off and extracted with methanol. The solutions containing SIZ-FITC (C_39_H_29_N_5_O_9_S_2_+, HRMS (*m*/*z*) 774.57; Fig. S1C) and SIZ-2FITC (C_60_H_41_N_7_O_14_S_3_^+^, HRMS (*m*/*z*) 1179.54; Fig. S1D) were collected and stored at 4 °C before use.

### 2.6 Development of signal-amplified FPA (SA-FPA)

**Fig. 1:**
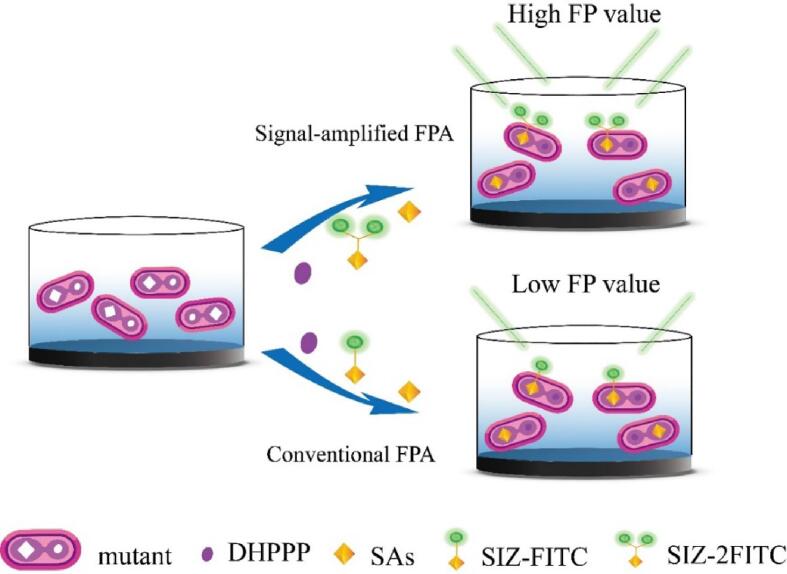
Schematic representation of the signal-amplified fluorescence polarization assay.

The assay principle of the SA-FPA was shown in Fig. 1. Briefly, the mutant was diluted with reaction buffer (containing DHPPP 4 mM and Mg^2+^ 40 mM, pH **=** 8.0), and the standard of SAs and fluorescent tracer were prepared by diluting the stock solution with deionized water. For each well, 50 μL of SAs solution, 50 μL of fluorescent tracer (1:400), and 50 μL of mutant (1:1000) were added, followed by incubation at 37 ℃ for 2 min. After that, the fluorescence polarization (FP) values were measured on a Synergy 2 multimode reader at λ_ex_ 485 nm and λ_em_ 528 nm.

**Fig. 2:**
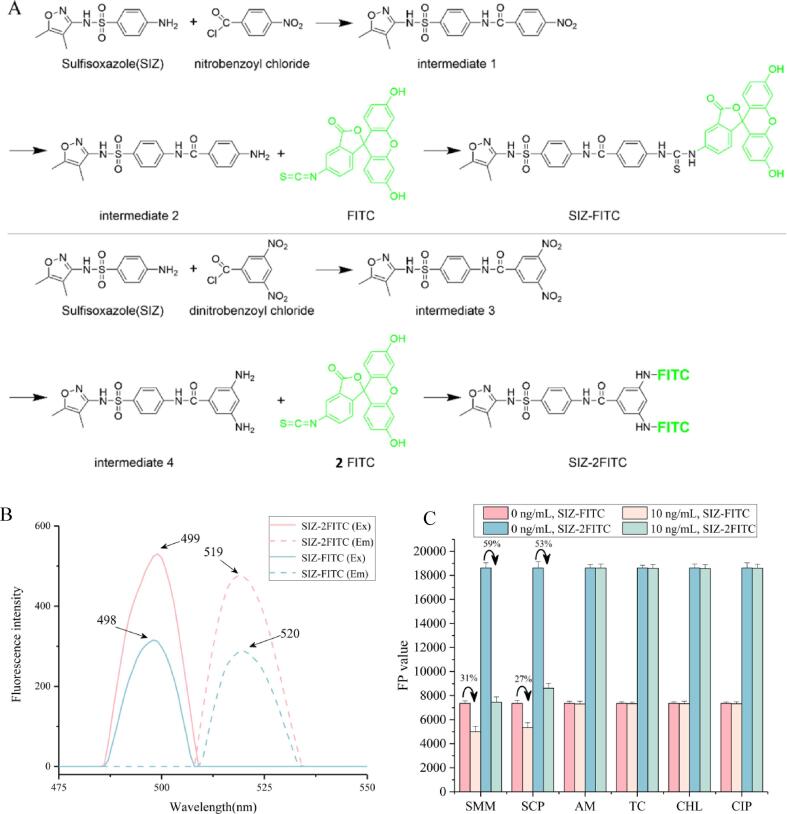
(A) Synthetic routes and (B) fluorescence spectra of two fluorescent tracers. (C) FP values for testing two representative SAs (SMM and SCP) and other drugs by using the two fluorescent tracers (mutant dilution 1:1000, tracer dilution 1:400, DHPPP 4 mM MgCl_2_ 40 mM, and incubation 5 min) (AM = amoxicillin, TC = tetracycline, CHL = chloramphenicol, CIP = ciprofloxacin, n = 5).

PABA (10 ng/mL) was used as the representative to optimize several important parameters, including the concentrations of mutant, fluorescent tracer, DHPPP, Mg^2+^ and the incubation time. After that, the 35 SAs were all tested by the SA-FPA method, and the related competitive curves were constructed by plotting the FP/FP_0_ (FP values of SAs at different concentrations divided by that of 0 ng/mL) against the SAs concentrations (log C). The 50% inhibition concentration (IC_50_) and the limit of detection (LOD) for 35 SAs were determined, where LOD was experimentally defined as the 10% inhibition concentration.

### 2.7 Method application and validation

The extraction of SAs from pork samples was as stated in our previous report (He, Cui, Liu, Feng, & Wang, 2022). Briefly, 1 g of homogenized pork and 5 mL of methanol were thoroughly mixed and centrifuged at 6000 g for 15 min by using a LG16-C centrifuger (Beijing Labcentrifuge Co., Ltd, Beijing China). Following that, 1 mL of the supernatant was dried with a nitrogen stream before being dissolved in 1 mL of PBS for analysis. For recovery studies, some blank pork samples were obtained from the controlled slaughterhouses, and 8 representative SAs were fortified into the blank samples (1, 10, and 50 ng/g) for extraction and analysis respectively. Finally, 50 unknown pork samples bought from several local markets were subjected to the SA-FPA and confirmed by our recently reported UPLC method (Wang, Wang, & Wu, 2022).

## 3 Results and discussions

### 3.1 Virtual mutation

**Fig. 3:**
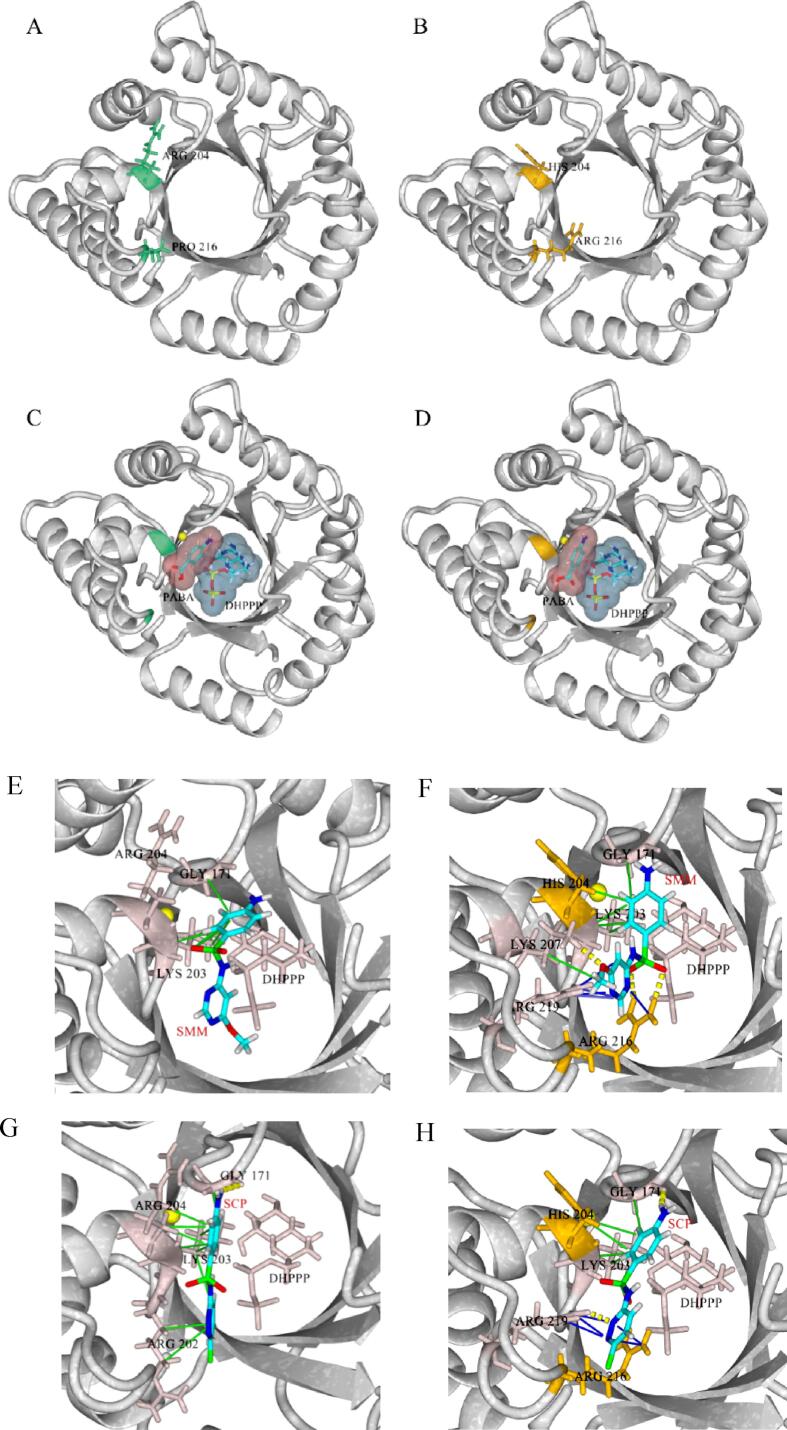
The 3D structures of (A) parental SaDHPS and (B) mutant, and the docking complexes of (C) parental SaDHPS/DHPPP/PABA and (D) mutant/DHPPP/PABA (The amino acids shown in green and orange were the mutated sites). Close up view of the docking complexes of (E) parental SaDHPS/DHPPP/SMM, (F) mutant/ DHPPP/SMM, (G) parental SaDHPS/DHPPP/SCP, and (H) mutant/DHPPP/SCP (The green line was hydrophobic interaction, the yellow dash-line was hydrogen bond, and the blue line was cation-Pi interaction). (For interpretation of the references to colour in this figure legend, the reader is referred to the web version of this article.)

In our recent study, a natural SaDHPS that can recognize 31 SAs was obtained, and the binding pocket and the key contact amino acids were identified (He, Liu, & Wang, 2021). For improving the affinity, 35 SaDHPS-SAs complexes were subjected to virtual mutation. The results predicted that when Arg204 was substituted with His and Pro216 was substituted with Arg, the protein energy decreased from −4340.02 kcal/mol to −4405.82 kcal/mol (ΔG = -65.80 kcal/mol). According to the YASARA’s principle, a negative ΔG value indicated the increased stability, which suggested the mutant had a more stable conformation. After virtual mutation, the binding energies for 35 SAs generally increased from 4.39 to 6.48 kcal/mol to 4.74–7.49 kcal/mol (Table 1), which indicated the success of evolution. For experimental proving its higher affinities, the IC_50_ for the 35 SAs when using the parental SaDHPS and the mutant were determined, and the results revealed the mutant was evolved successfully (Table 2). As shown in Fig. 3A and 3B, the 3D conformation of the mutant was identical with that of the parental SaDHPS, and the binding of DHPPP with the mutant was consistent with that of the parental SaDHPS (Fig. 3C and 3D). These results indicated that the spatial formation of the binding pocket was not changed after mutation, and this provided the base for the subsequent molecular docking. Therefore, these two positions were selected as mutagenesis sites to produce the SaDHPS mutant.

**Table 1:** Docking results of the SaDHPS mutant with 35SAs. The red atoms, blue dots and red circle were the binding sites of hydrophobic interactions, hydrogen bonds and Cation-Pi interactions respectively. The values in parentheses were the binding energies of parental SaDHPS for SAs from our previous report (He et al., 2021).

Drug	Molecular structure	Binding energy (kcal/mol)	Contact amino acid		
			Hydrogen bond	Hydrophobic interaction	Cation-Pi
PABA		4.62 (4.0)	Gly171 Arg202	Gly171Lys203 His204	---
SN		5.16 (4.43)	Gly171	Gly171 Lys203 His204	---
SA		5.42 (5.03)	Gly171 Arg219	Gly171 Lys203 His204	---
SG		5.47 (5.21)	Gly171 His214 Arg202	Gly171 Phe172	---
DCS		5.33 (4.92)	Arg219	Lys203 His204	---
MBS		4.74 (4.39)	Arg216	Gly171 Lys203 His204	---
SMZ		5.98 (5.2)	Gly171 Arg216 Arg202	Gly171 Lys203 His204	Arg219(N)
SIZ		5.64 (4.97)	Gly171 Arg219	Gly171 Arg202 Lys203	Arg216(N) Arg219(O)
SMT		5.71 (5.48)	Gly171 Arg216	Gly171 Lys203 His204	---
STZ		5.86 (5.36)	Gly171 Arg216	Gly171 Lys203 His204	Arg216
SXL		5.96 (5.57)	Gly171 Arg216	Gly171 Lys203 His204 Phe172	Arg219
SPA		6.68 (5.58)	Gly171 Arg216	Gly171 Lys203 His204	---
BSN		6.45 (6.27)	Arg216	Gly171 Lys203 His204	Arg216
AS		5.92 (5.80)	Arg219	Gly171 Lys203 His204	Arg216
SB		5.73 (5.58)	Gly171 Arg219	Gly171 Lys203 His204	---
SD		6.12 (4.73)	Arg216	Gly171 Lys203 His204	Arg216
SCZ		5.63 (5.91)	---	Gly171 Lys203 His204 Lys207	Arg216 Arg219
SM_2_		5.47 (5.62)	Arg204	Gly171 Lys203 His204	---
SMP		5.98 (5.67)	Gly171 Arg216 Lys207	Gly171 Lys203	Arg216 Arg219
SMM		5.93 (5.54)	Arg216 Lys207	Gly171 Lys203 His204 Lys207	Arg216 Arg219
SQ		6.8 (5.3)	Gly171 Arg216	Gly171 Lys203 His204	---
SCP		5.63 (5.49)	Gly171 Arg219	Gly171 Lys203 His204	Arg216 Arg219
SDX		6.09 (5.53)	Gly171 Arg216 Lys207	Gly171 Lys203 His204	Arg216 Arg219
SPD		6.23 (5.29)	Arg216 Lys207	Gly171 Lys203 His204	Arg216 Arg219
SIM		6.05 (5.08)	Arg216 Arg219	Gly171 Lys203 His204	Arg216 Arg219
SMD		6.04 (5.95)	Gly171 Arg216 Lys207	Gly171 Phe172 Lys203 His204	Arg216 Arg219
SMR		5.64 (5.43)	Arg216	Gly171Lys203 His204	---
SPY		5.93 (5.86)	Gly171 Arg219	Gly171 Lys203 His204 Arg216	Arg216 Arg219
SL		5.83 (5.64)	Arg216 Arg219 Val204	Gly171 Lys203 His204	Arg219
SEP		6.13 (4.84)	Gly171 Arg216	Gly171 Lys203 His204	Arg216 Arg219
SZ		6.22 (5.82)	Gly171 Arg202	Gly171 Lys203 His204	---
SSZ		7.49 (6.47)	Arg216	Gly171 Lys203 His204	Arg216 Arg219
PST		6.95 (6.48)	Gly171 Arg202	Gly171 Lys203 His204	---
ASM		6.12 (6.10)	Gly171 Lys207	Gly171 Lys203 His204	Arg219
ASZ		6.18 (6.12)	Arg219	Thr51 Gly171 Phe172 Lys203 His204Arg216	Arg216 Arg219
SSA		6.20 (5.39)	---	Gly171 Lys203 His204	---

**Table 2:** Determination parameters of the FPA method for 35 SAs when using different reagent combinations.

drug	mutant + SIZ-FITC		SaDHPS + SIZ-2FITC		mutant + SIZ-2FITC	
	IC_50_ (ng/mL)	LOD (ng/mL)	IC_50_ (ng/mL)	LOD (ng/mL)	IC_50_ (ng/mL)	LOD (ng/mL)
PABA	26.81	1.14	14.48	0.52	8.08	0.281
SSZ	7.42	0.31	3.47	0.1	2.09	0.057
SXL	7.42	0.24	4.97	0.11	2.16	0.030
SL	8.47	0.44	5.05	0.14	2.96	0.063
AS	9.11	0.44	5.17	0.21	3.08	0.142
PST	9.88	0.36	6.25	0.21	3.32	0.113
STZ	10.17	0.39	6.55	0.21	3.65	0.141
BSN	11.99	0.42	8.66	0.23	4.47	0.150
SDX	12.58	0.47	9.45	0.26	5.06	0.144
SN	12.86	0.66	9.63	0.4	5.22	0.238
SCZ	13.48	0.72	6.02	0.37	5.54	0.208
ASM	14.02	0.82	10.01	0.4	5.98	0.236
SZ	14.07	1.06	10.43	0.66	6.02	0.250
SMR	16.25	1.13	10.48	0.63	6.03	0.321
SB	19.24	1.25	10.72	0.58	6.11	0.369
SM_2_	20.02	0.55	6.88	0.52	6.21	0.194
SMT	20.95	1.33	11.02	0.68	6.88	0.351
SPD	22.34	1.1	14.59	0.71	7.20	0.287
SMM	25.04	1.41	12.00	0.76	7.41	0.379
SSA	25.41	1.85	11.65	0.82	7.70	0.414
SD	26.16	1.53	16.20	0.8	8.04	0.382
SMD	26.42	1.22	14.21	0.53	8.05	0.347
SA	27.09	1.48	14.61	0.61	8.29	0.387
SPY	28.16	1.66	15.10	0.75	9.32	0.466
ASZ	28.44	1.74	15.34	0.82	9.33	0.400
SCP	30.57	2.16	16.60	0.91	9.94	0.495
SPA	31.98	1.7	20.17	0.98	10.46	0.389
SG	33.11	2.51	19.40	0.98	11.89	0.581
SMP	33.21	3.25	19.45	1.43	11.90	0.783
SIZ	34.59	2.68	20.06	1.51	12.46	0.696
SQ	35.19	4.25	25.70	1.64	13.69	0.859
SMZ	37.23	2.64	20.67	1.69	14.03	0.575
DCS	40.12	5.31	25.23	1.84	14.35	0.667
SEP	42.60	5.72	26.51	1.93	14.38	0.890
SIM	44.77	4.33	25.49	2.21	14.97	0.711
MBS	62.83	7.74	35.74	2.52	19.66	1.116

### 3.2 Intermolecular interaction mechanisms of mutant and SAs

PABA was firstly docked with the mutant. As shown in Fig. 3C and 3D, PABA fitted well with the binding pocket, whose location was almost same as that in parental SaDHPS. However, the binding energy and the number of contacting amino acids all increased, and the binding sites expanded to its primary amine (Table 1), indicating the improved affinity. Then the 35 SAs were docked with the mutant respectively, and the results showed all the 35 SAs could enter the binding pocket (Fig. S2). Compared with the parental SaDHPS, the numbers of amino acids contacting with the 35 SAs all increased (Table 1), and this could be seen from the representative docking complexes with SMM and SCP shown in Fig. 3E-3H. As shown in Fig. 3F and 3H, Gly171, Lys203, His204 and Arg216 were the key contacting amino acids that constructed the binding pocket for SAs.

As shown in Table 1, the mutant formed strong hydrophobic interactions and hydrogen bonds with all the 35 SAs. Hydrophobic interactions were generally formed between the phenyl ring of SAs and Gly171, Lys203, and His204, while hydrogen bonds were generally formed between the primary amine and -SO_2_N- part of SAs and Gly171 and Arg216. These docking results were similar to that of parental SaDHPS which mainly interacted with the SAs core structure via hydrophobic interactions and hydrogen bonds.

However, cation-Pi interactions appeared in the bindings with 21 SAs via Arg216 and Arg219, and the binding sites were located at their side chains (Table 1). This revealed the mutant could interact with both of their core structure and side chains. In contrast, cation-Pi interactions in the bindings of parental SaDHPS with SAs were negligible. In combination of the binding energies, the numbers of contacting amino acid, the newly appeared cation-Pi interaction and the enlarged binding sites, it could be said the mutant achieved higher affinities for the 35 SAs than the parental SaDHPS. As the results, the mutant showed higher sensitivities for these SAs than the parental SaDHPS (Table 2).

### 3.3 Characterization of SaDHPS mutant

According to the result of the multipoint mutant, the amino acid sequence of the mutant was deduced. As shown in Fig. S3, the amino acid sequences of the parental SaDHPS and the mutant were identical except the two mutagenesis sites, which were outside the 100% conserved region. As shown in Fig. S4a, the PCR result indicated that the target gene of 804 bp was amplified from the recombinant plasmid, matching the size of mutant gene. The double digestion assay showed that the mutant gene (804 bp) and pET-32a (5900 bp) were obtained from the recombinant plasmid (Fig. S4b), indicating the expression vector was constructed successfully. The parental SaDHPS was a soluble protein with a size of 30 kDa (He, Liu, & Wang, 2021), and the present SDS-PAGE result indicated that the mutant was also a soluble form (Fig. S4B). Meanwhile, the western blotting result showed that the molecular weight of the mutant was about 45 kDa (Fig. S4C), which was because the mutant was a fusion containing some tags, including 6 × HIS-tag, TRX-tag, and S-tag (total 15 kDa). From the above results, it could be said the SaDHPS mutant was obtained.

The instability and degradation of the receptor *in vitro* restricted its practical application. For evaluation of the stabilities of parental SaDHPS and SaDHPS mutant, two receptors were placed at −20 ℃ for 6 months and at 37 ℃ for 7 days, respectively. At the indicated times, each receptor was taken to incubate with SIZ-FITC, and the FP values were measured. As shown in Fig. S5, the signals from the parental SaDHPS decreased 72% after stored at −20 °C for 6 months and decreased 88% after stored at 37 °C for 7 days. Meanwhile, the signals from the SaDHPS mutant only decreased 36% after stored at −20 °C for 6 months and decreased 51% after stored at 37 °C for 7 days. Collectively, the stability of SaDHPS mutant was higher than that of parental SaDHPS.

### 3.4 Characterization and comparison of the two fluorescent tracers

In the present study, an enhanced fluorescent tracer and a conventional fluorescent tracer were synthesized. The results of ESI-HRMS (Fig. S1) indicated that the two fluorescent tracers were synthesized successfully. As shown in Fig. 2B, the excitation wavelengths and the emission wavelengths of SIZ-FITC (λ_ex_ 498 nm/λ_em_ 520 nm) and SIZ-2FITC (λ_ex_ 499 nm/λ_em_ 519 nm) were almost identical, indicating the two tracers had comparability.

For comparison of the two fluorescent tracers, the mutant-based FPA experiments were conducted by using two representative SAs (SMM and SCP - at concentrations of 0 and 10 ng/mL) and the two fluorescent tracers (SIZ-FITC and SIZ-2FITC - at the same concentration of dilution 1: 400). The FP values and inhibition ratios (1-FP/FP_0_) were used to assess the workability of two fluorescent tracers. The results showed that when the concentration of SAs was 0 ng/mL, the FPA experiments when using SIZ-2FITC obtained high FP values (about 2.6-fold) in comparison with the use of SIZ-FITC (Fig. 2C). These findings indicated that the mutant could bind with both tracers for the development of FPA method, and the signal amplification of SIZ-2FITC was observed. Furthermore, when adding 10 ng/mL of SAs, it was obvious that the inhibition ratios when using SIZ-2FITC (59% for SMM and 53% for SCP) were higher than that when using SIZ-FITC (31% for SMM and 27% for SCP), indicating the higher sensitivity was obtained when using such enhanced fluorescent tracer. Accordingly, SIZ-2FITC was selected for the development of SA-FPA. Meanwhile, four other classes of drugs, including amoxicillin, tetracycline, chloramphenicol, and ciprofloxacin, were also tested to evaluate the specificity of the mutant. As shown in Fig. 2C, the FPA method showed negligible inhibition ratios (<1 %) to these drugs, indicating the mutant could specifically recognize SAs.

### 3.5 Optimization of the SA-FPA

For the establishment of an SA-FPA method, a couple of essential parameters that influenced the sensitivity were optimized. Firstly, the working concentrations of mutant and SIZ-2FITC were optimized. The checkerboard assay result showed that the optimal dilution of the mutant was 1:1000 and that of SIZ-2FITC was 1:400 (Fig. S6A). Secondly, several co-factors of the reaction buffer, including DHPPP and Mg^2+^, were further optimized. It could be seen from Fig. S6B and S6C that the highest inhibition ratios were obtained when the concentrations of DHPPP and MgCl_2_ were 4 mM and 40 mM, respectively. Finally, the competition time was determined to be two minutes because the inhibition rate reached constant after two minutes of incubation (Fig. S6D). Taken together, the above five parameters (1:1000 mutant, 1:400 SIZ-2FITC, 4 mM of DHPPP, 40 mM of MgCl_2_, and incubating for 2 min) were used for the subsequent work.

### 3.6 Method performance

After optimization, the 35 SAs were spiked into the blank pork samples with different concentrations (1, 10, 50 ng/g) to be extracted and assayed by the SA-FPA method, respectively. As summarized in Table 2, the IC_50_ values for the 35 drugs ranged from 2.09 to 19.66 ng/mL, and the LODs were in the range of 0.03–1.16 ng/mL. In comparison, the LODs of the 35 SAs when using the SIZ-FITC were in the range of 0.24–7.74 ng/mL. This meant that the sensitivities for the 35 SAs were improved for 2.8-fold (BSN) to 8.6-fold (DCS) by using SIZ-2FITC. The representative calibration curves of SMM shown in Fig. S7 just proved the sensitivity improvement effect. Meanwhile, the LODs of the 35 SAs when using the parental SaDHPS and SIZ-2FITC were 0.1–2.52 ng/mL (Table 2). This meant that the sensitivities for the 35 SAs were improved for 1.5-fold (SCZ) to 3.7-fold (SXL) when using the mutant, proving the parental SaDHPS was evolved successfully.

**Fig. 4:**
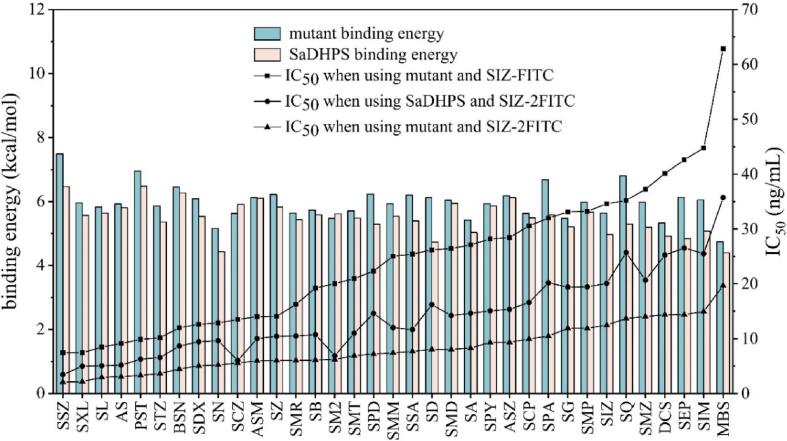
Overview of the binding energies of the parental SaDHPS and the mutant for the 35 SAs, and IC_50_ for the 35 SAs when using the mutant + SIZ-FITC, parental SaDHPS + SIZ-2FITC, and mutant + SIZ-2FITC.

In the present SA-FPA method, the SaDHPS mutant and enhanced fluorescence tracer were the critical factors determining the detection spectra and sensitivity. For comprehensive demonstration of the evolution of SaDHPS and the effectiveness of the enhanced fluorescence tracer, the IC_50_ of 35 SAs in three different combinations were integrated in Fig. 4. It could be seen that the use of the SaDHPS mutant generally resulted in lower IC_50_ values than using the parental SaDHPS, indicating the parental SaDHPS was evolved successfully. Meanwhile, the IC_50_ for the 35 SAs when using the enhanced fluorescence tracer were generally lower than that when using the conventional fluorescent tracer containing one fluorophore molecule. This finding suggested that the use of such enhanced fluorescence tracer could improve the sensitivity of a FPA method.

### 3.7 Method application

For evaluation of the accuracy and precision of the developed SA-FPA, 8 representative SAs were spiked into the blank pork samples and assayed. As shown in Table S1, the inter-assay recoveries of the 8 SAs were from 70.8% to 86.6%, and the intra-assay recoveries were from 71.3% to 88.4%, with the coefficients of variation of 7.1%-17.7%. As shown in Fig. S7, the calibration curve of matrix matched SMM was identical with that of SMM standard, regardless of the used fluorescent tracer, which indicated the matrix effect was negligible. So the determination of these SAs in pork sample were according to their standards. Finally, 50 real pork samples were detected by present SA-FPA and confirmed by a UPLC method. According to the SA-FPA results, one sample was found to be positive, and the others were negative. The UPLC result verified that the positive sample contained SM_2_ (5.03 ng/g), and other samples were confirmed as negative. Therefore, the present SaDHPS mutant-based SA-FPA could be used as a rapid, sensitive, and practical tool for multi-screening of the residues of the 35 SAs in large number of pork samples.

### 3.8 Comparison with the related methods

Up to now, six fluorescence polarization immunoassays (FPIA) (Eremin et al., 2005, Eremin et al., 1994, Murtazina et al., 2004, Wang et al., 2007, Wang et al., 2008, Chen et al., 2014) and two DHPS-based fluorescence polarization assays (FPA) (He et al., 2021, Wang et al., 2015) were reported for the detection of SAs. For comparison with the present method, some essential parameters of those methods were summarized in Table S2. First, this study for the first time developed a signal-amplified FPA for SAs detection based on a novel fluorescent tracer containing two fluorophore molecules. Second, the detection spectrum of the present method was broader than all of those methods. Third, the sensitivity of this method was higher than all of those methods. Fourth, the assay time was comparable to our previous FPA method but shorter than other methods. Overall, the present signal-amplified FPA showed generally better performances than the previously reported FPIAs and DHPS-based FPAs.

## 4 Conclusion

The fluorescence polarization immunoassay is a simple and rapid method for multi-screening of SA residues in animal-derived food. However, the detection spectrum and the sensitivity of conventional FPIA are not satisfactory. In this study, we for the first time produced a mutant of SaDHPS by using the multipoint mutagenesis technique and developed a signal-amplified FPA for multi-detecting 35 SAs based on an enhanced fluorescent tracer containing two fluorophore molecules. The results demonstrated that the mutant had improved affinities to SAs, and the method sensitivities were improved for 2.8–8.6 folds compared with the conventional fluorescent tracer containing one fluorophore molecule. The present method could be used for multi-screening the residue of 35 SAs in large-scale pork samples. Furthermore, this method provides a novel idea and a promising strategy for improving the sensitivity of other fluorescent methods.

## Compliance with ethical standards

All of the authors declare that they have no conflict of interest.

## CRediT authorship contribution statement

**Tong He:** Methodology, Writing – review & editing. **Peng Lei Cui:** Methodology. **Shuai Zhang:** Methodology, Validation. **Yu Hang Fan:** Validation. **Qiu Shi Jin:** Validation. **Jian Ping Wang:** Funding acquisition, Conceptualization, Writing – review & editing.

## Declaration of Competing Interest

The authors declare that they have no known competing financial interests or personal relationships that could have appeared to influence the work reported in this paper.

## Data Availability

Data will be made available on request.
