# Supplementary material for: Development of a receptor based signal amplified fluorescence polarization assay for multi-detection of 35 sulfonamides in pork
Source: Food Chem X. 2023 Sep 6;19:100867. doi: 10.1016/j.fochx.2023.100867 (PMC10534214; doi:10.1016/j.fochx.2023.100867)
Supplement: Supplementary data 1 [file mmc1.docx]

Supplementary Materials for

**Development of a receptor based signal amplified** **fluorescence polarization assay for multi-detection of 35 sulfonamides in pork**

Tong He ^a,^ ^[[1]](#footnote-1)^, Peng Lei Cui ^b 1^, Shuai Zhang ^a^, Yu Hang Fan ^a^, Qiu Shi Jin ^a^, Jian Ping Wang ^a^ ^[[2]](#footnote-2)^*

^a^ College of Veterinary Medicine, Hebei Agricultural University, Baoding Hebei 071000, China

^b^ College of Science, Hebei Agricultural University, Baoding Hebei 071000, China

Figure S1. The mass spectrometry results of (A) intermediate 2, (B) intermediate 4, (C) SIZ-FITC, and (D) SIZ-2FITC.


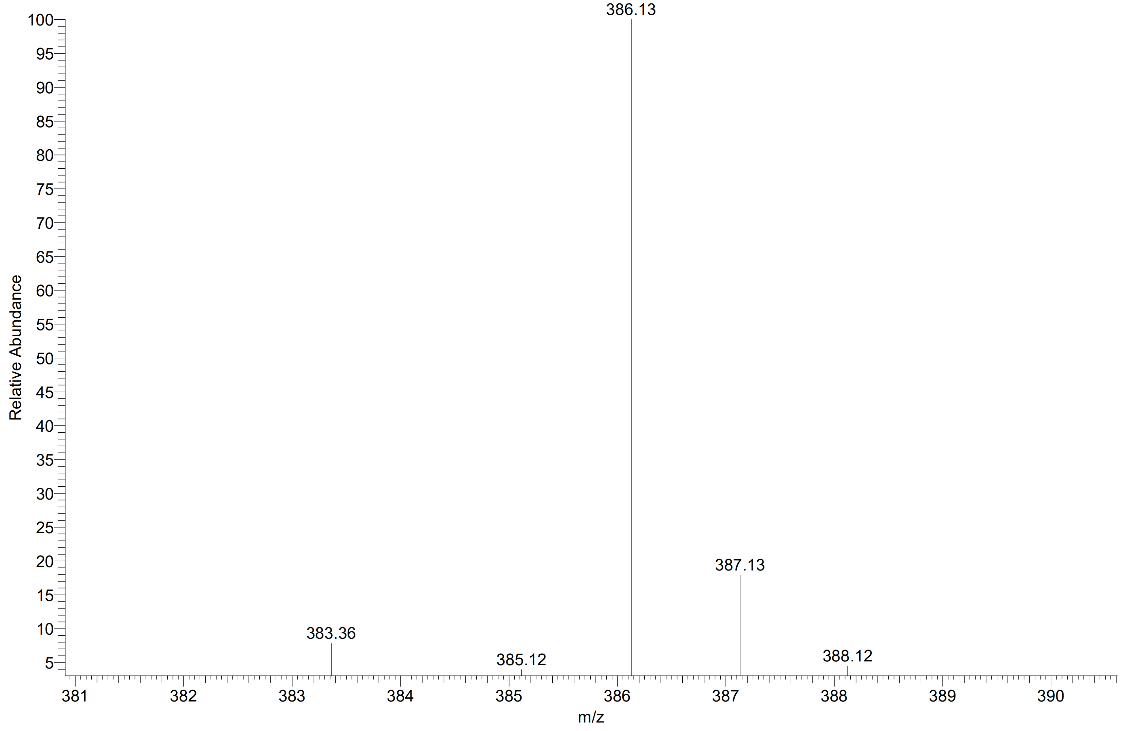


A


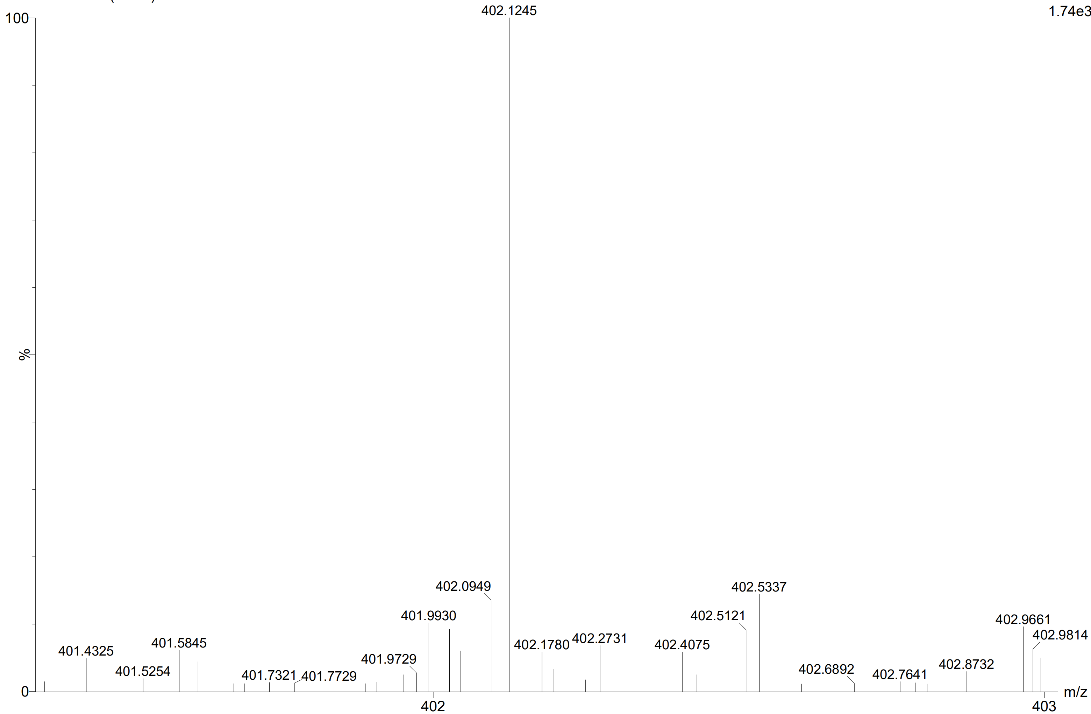


B


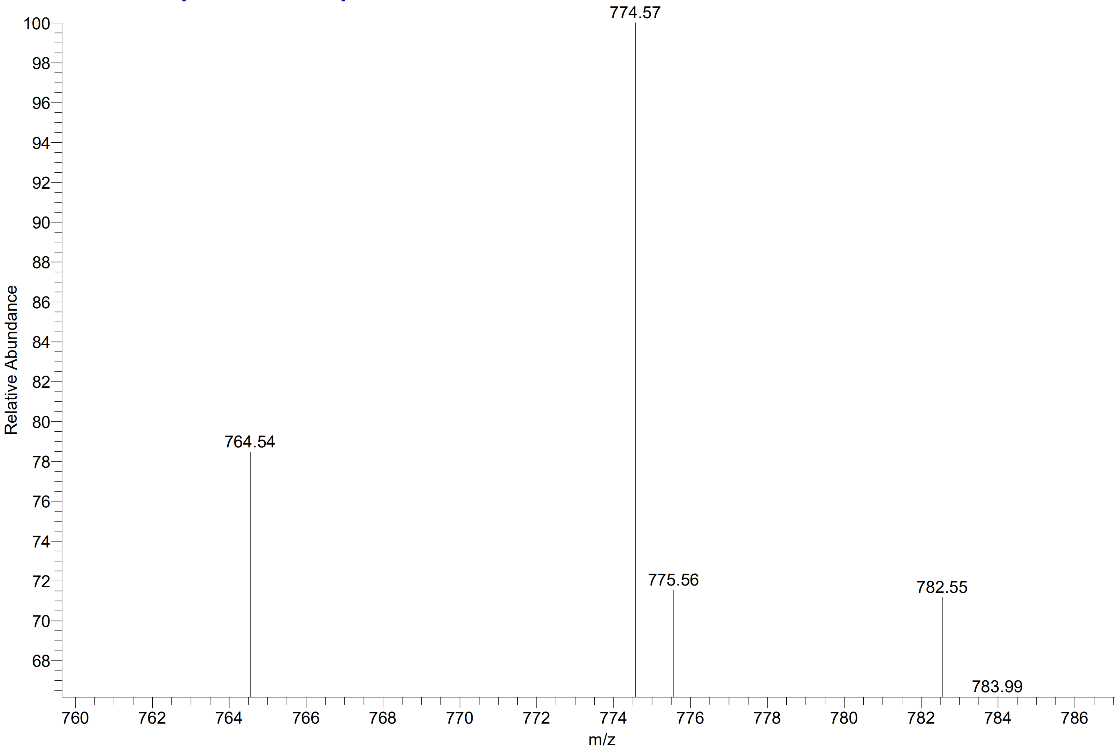


C


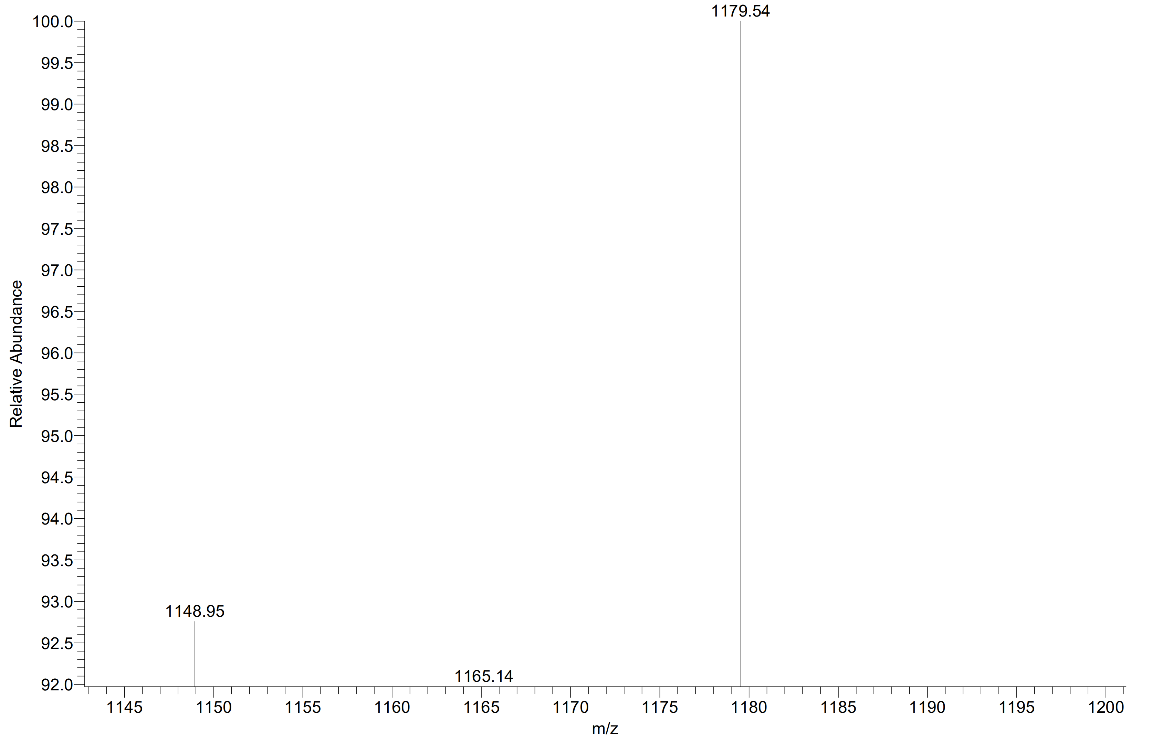


D

Figure S2. Docking complexes of the mutant with 35 SAs.


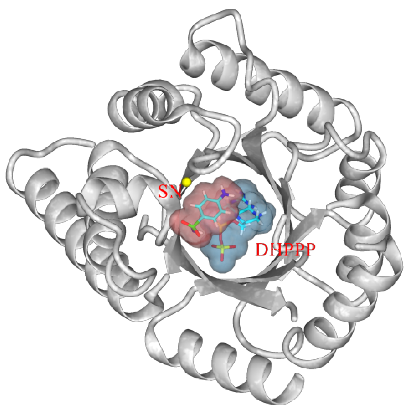

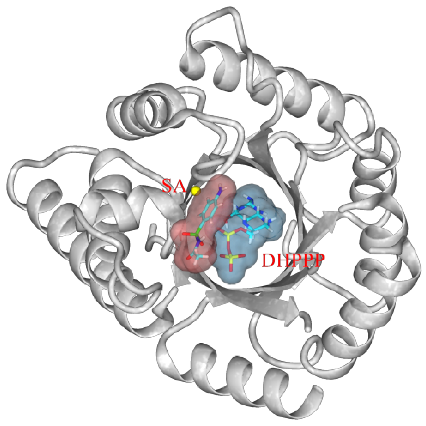

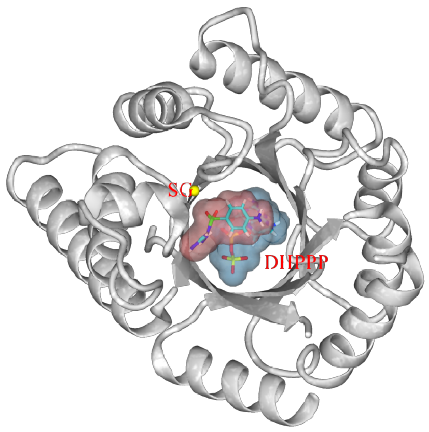

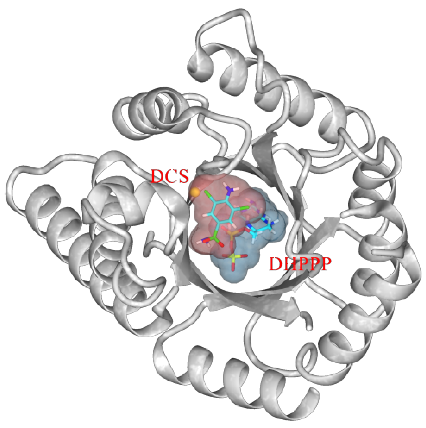

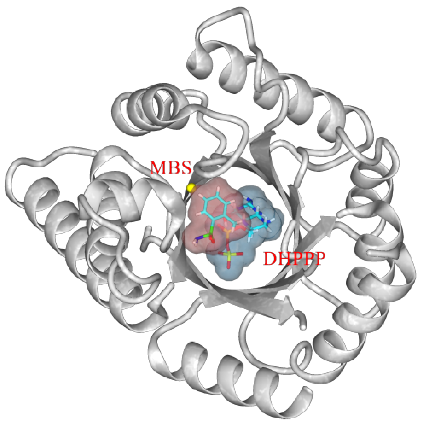

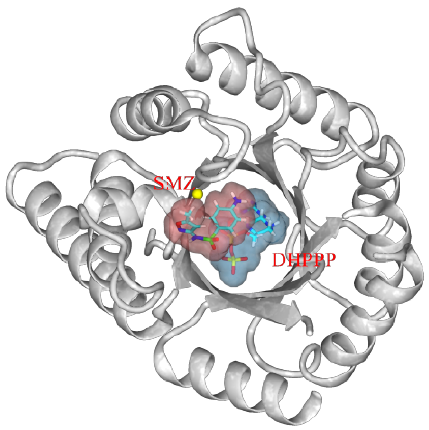


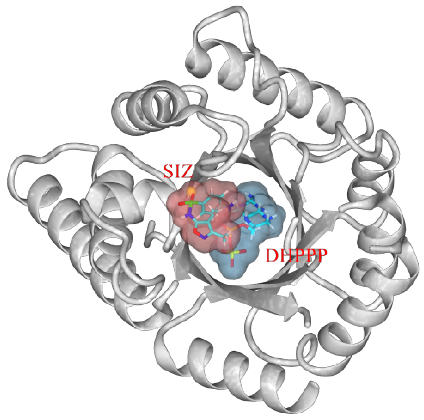

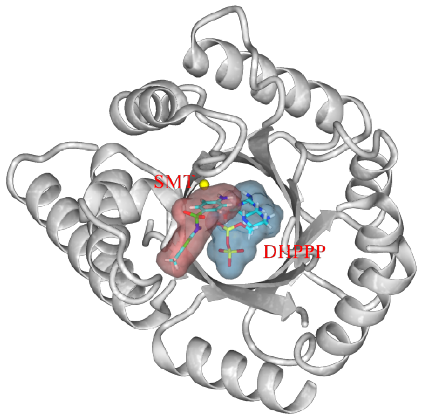

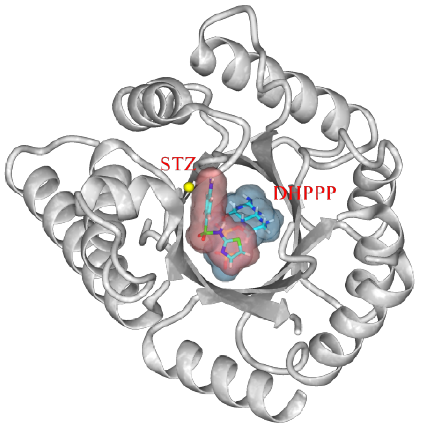


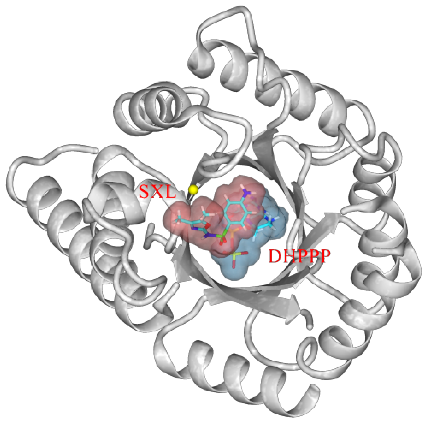

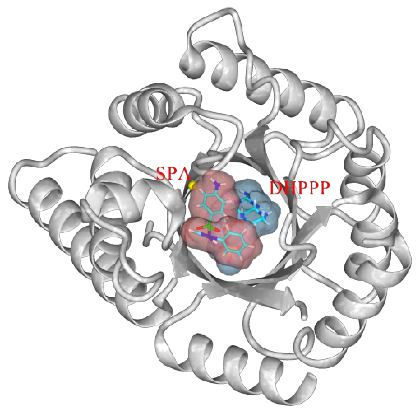

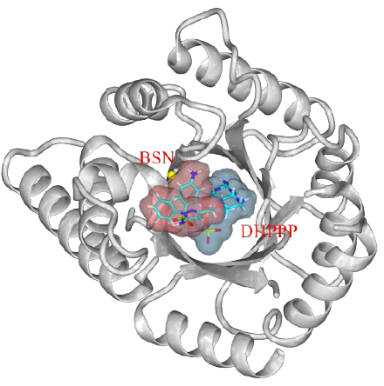


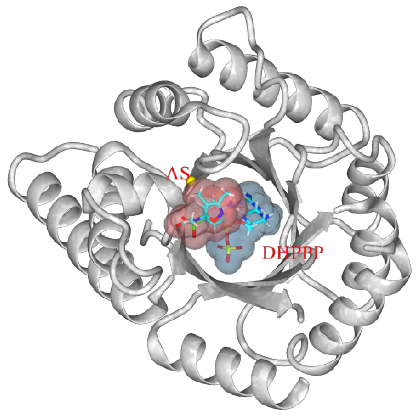

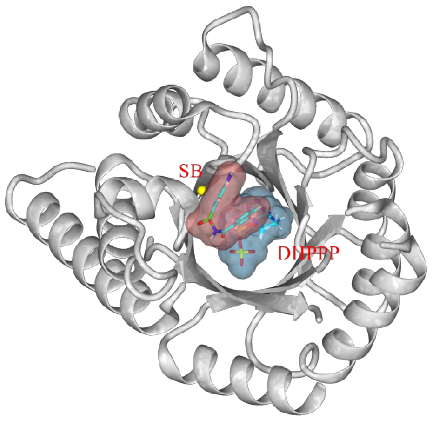

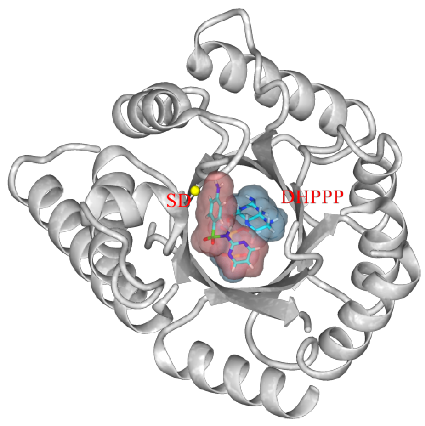


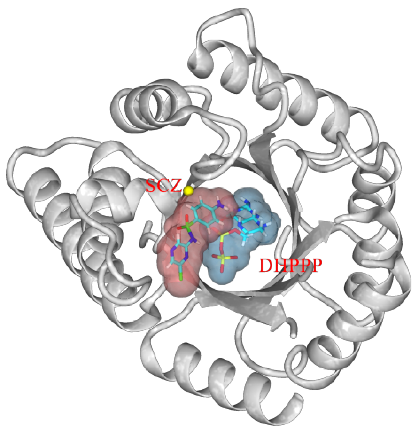

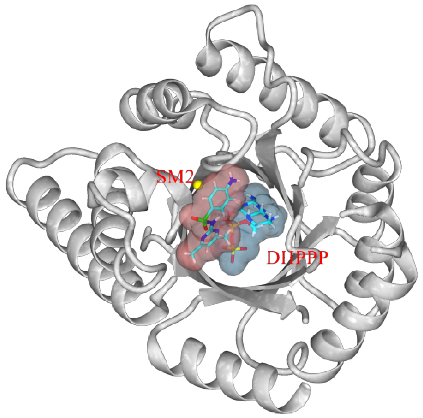

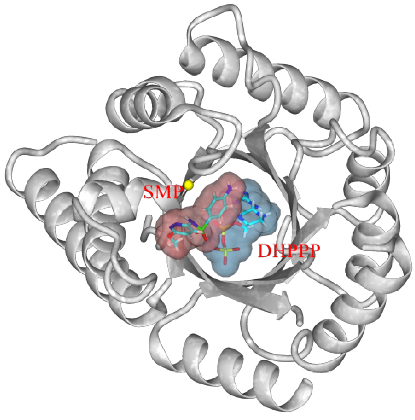

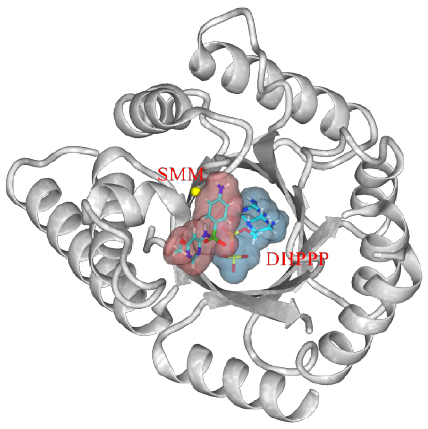

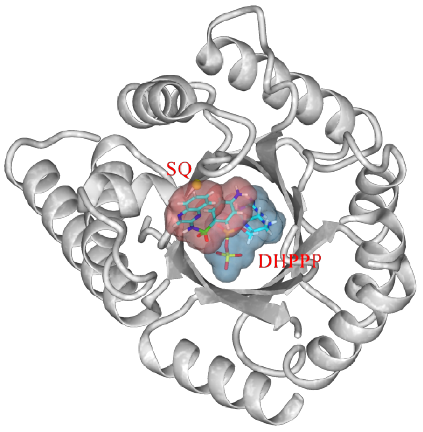

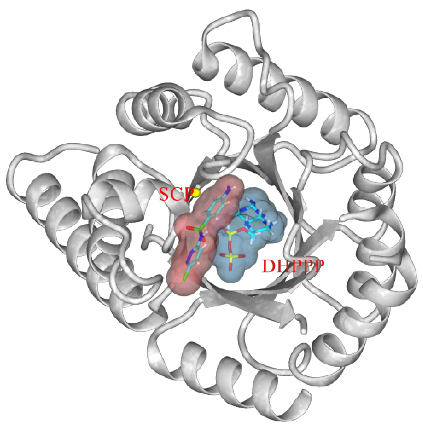

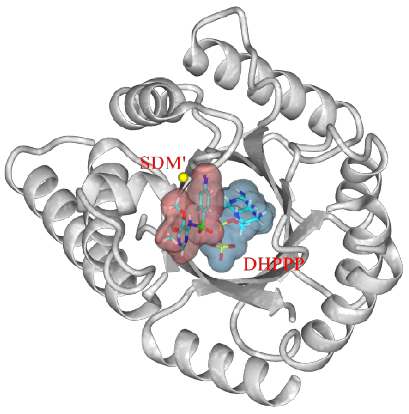

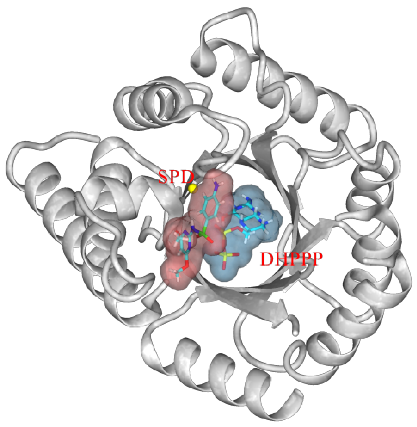

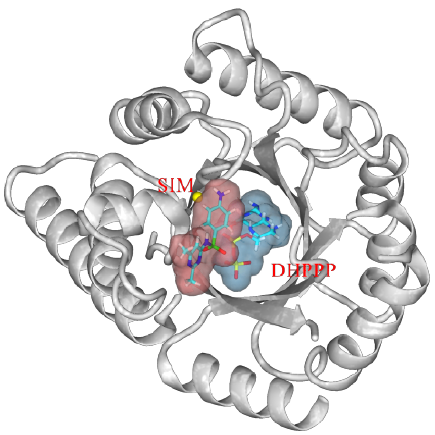

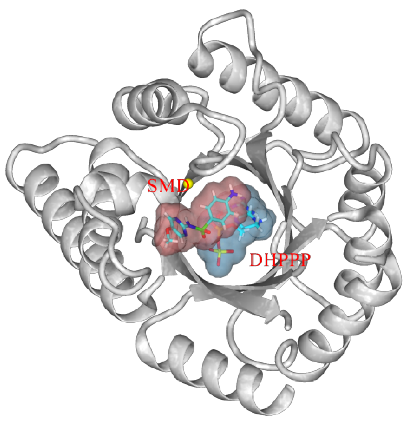

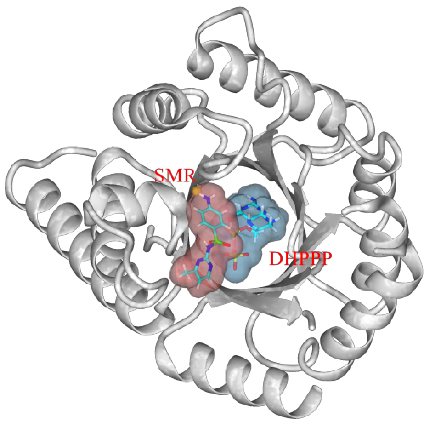

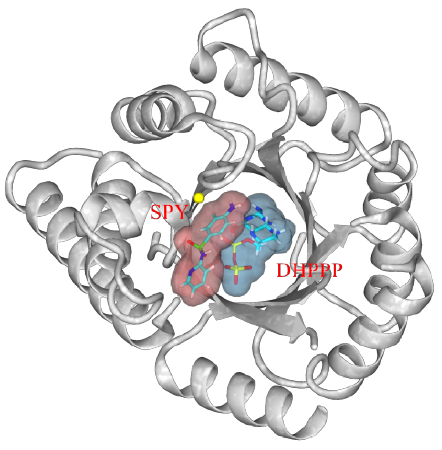

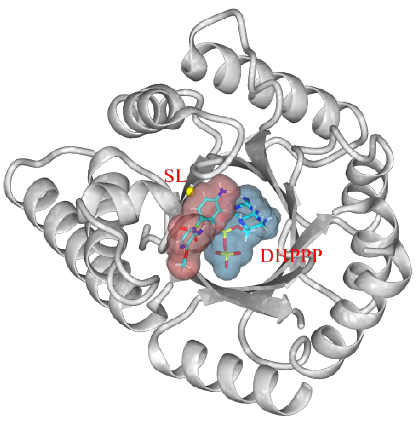

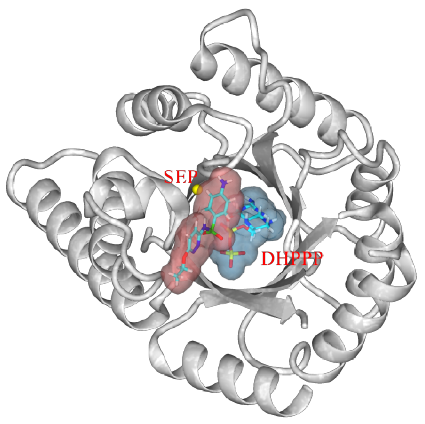

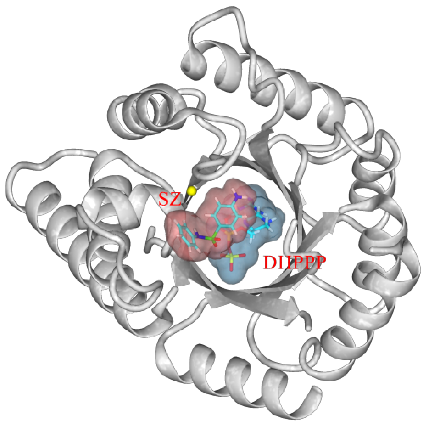


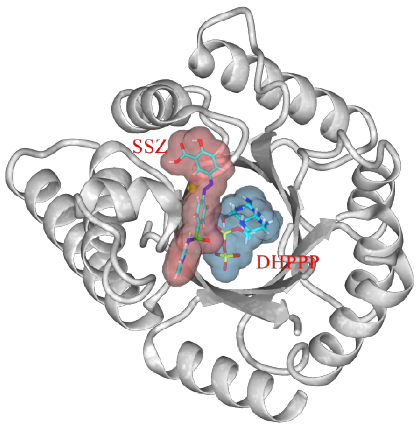

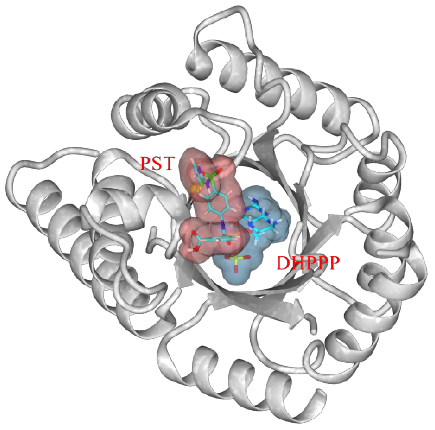

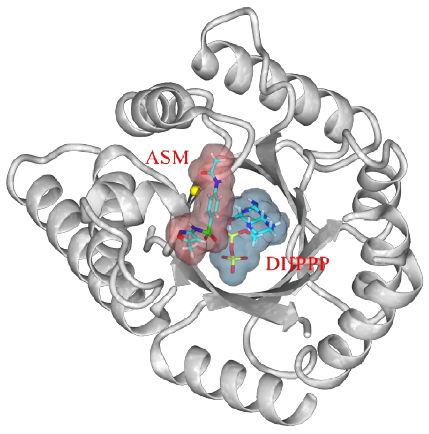

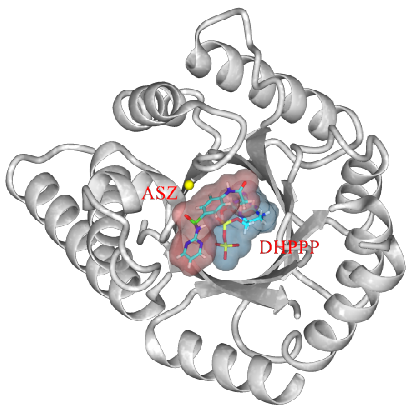

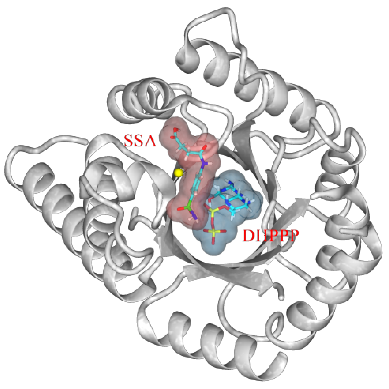


Figure S3. Comparison of the amino acid sequences of the parental SaDHPS and the mutant (GenBank ID: Z84573.1, PDB ID: 1AD1). The amino acids highlighted in red are the mutation sites. The amino acids highlighted in dark blue are the 100% conserved residues.


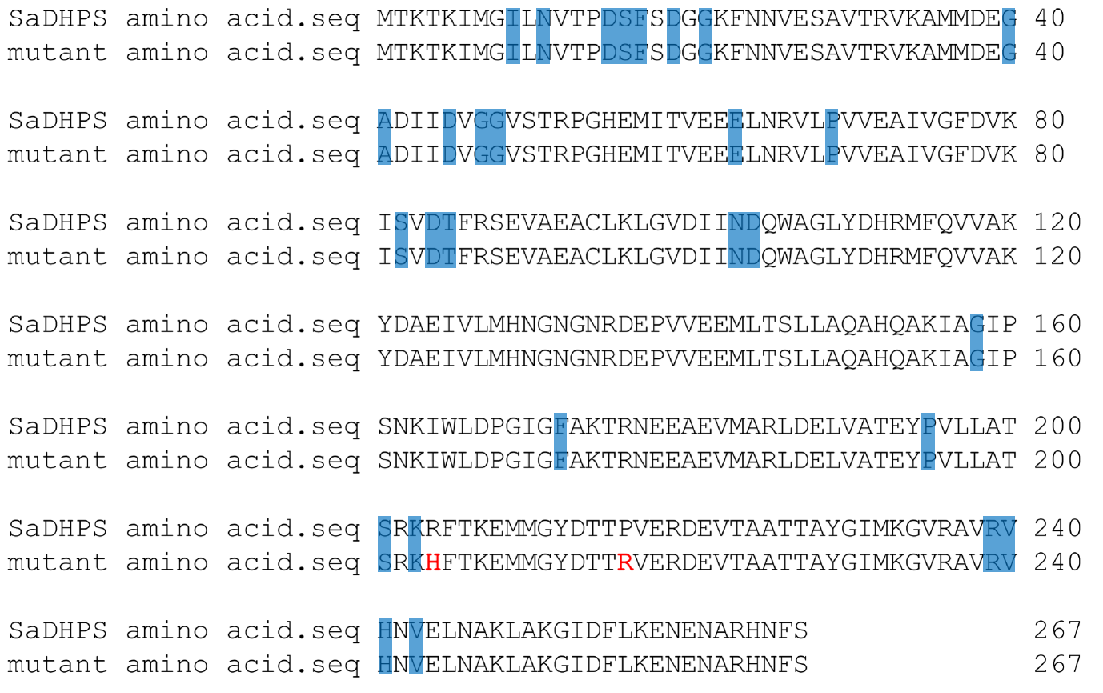


Figure S4. (A) Characterization results of express vector pET32a-mutant by (a) PCR and (b) double digestion. (B) SDS-PAGE results of the mutant (lane 1, supernatant, lane 2, purified supernatant, lane 3, inclusion body, lane 4, purified inclusion body). (C) Western blotting result of the mutant.


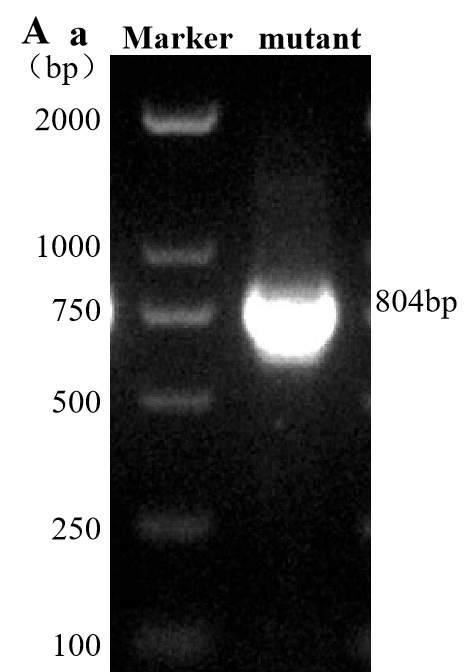

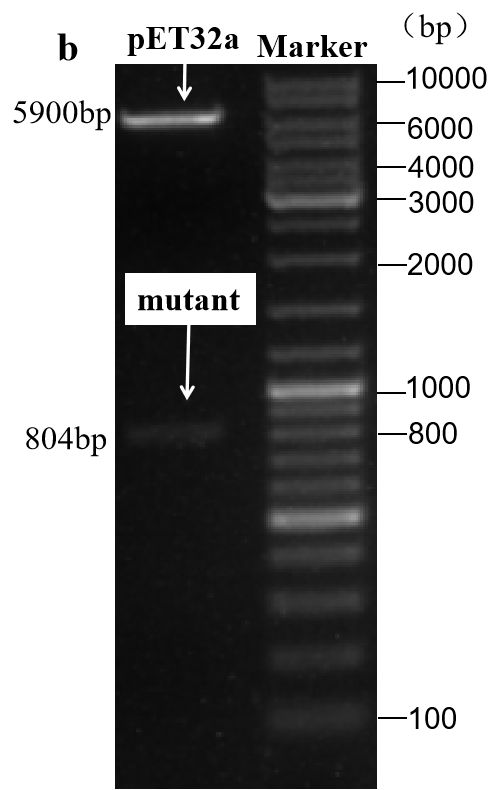


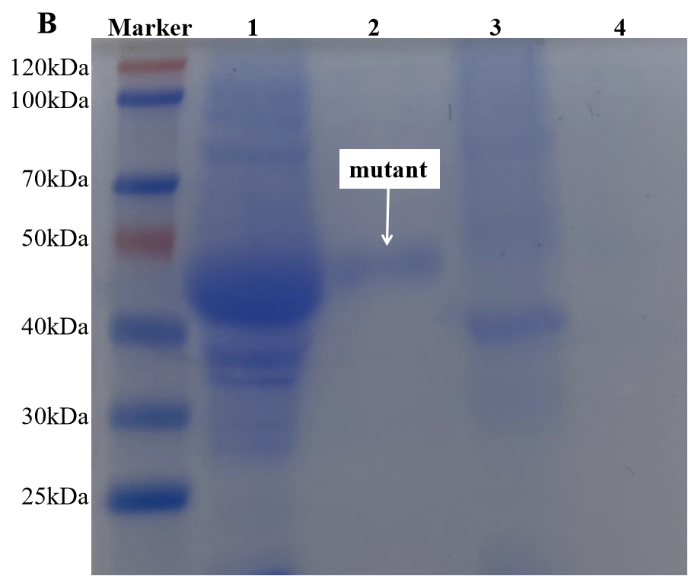

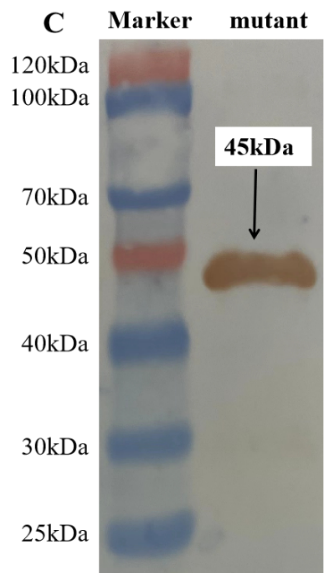


Figure S5. Signals of parental SaDHPS and SaDHPS mutant after stored at -20 °C for 6 months (A) and at 37 °C for 7 days (B). Residual activity (%) = FP value (after stored) / FP value (before stored) × 100%.


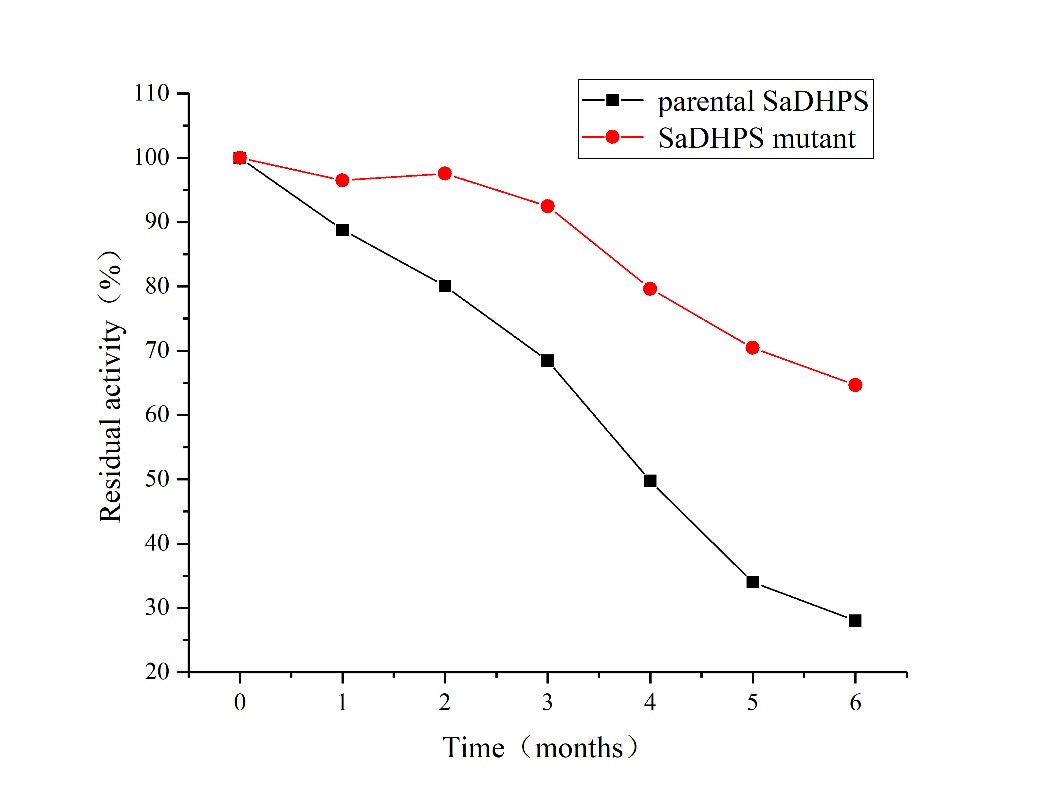

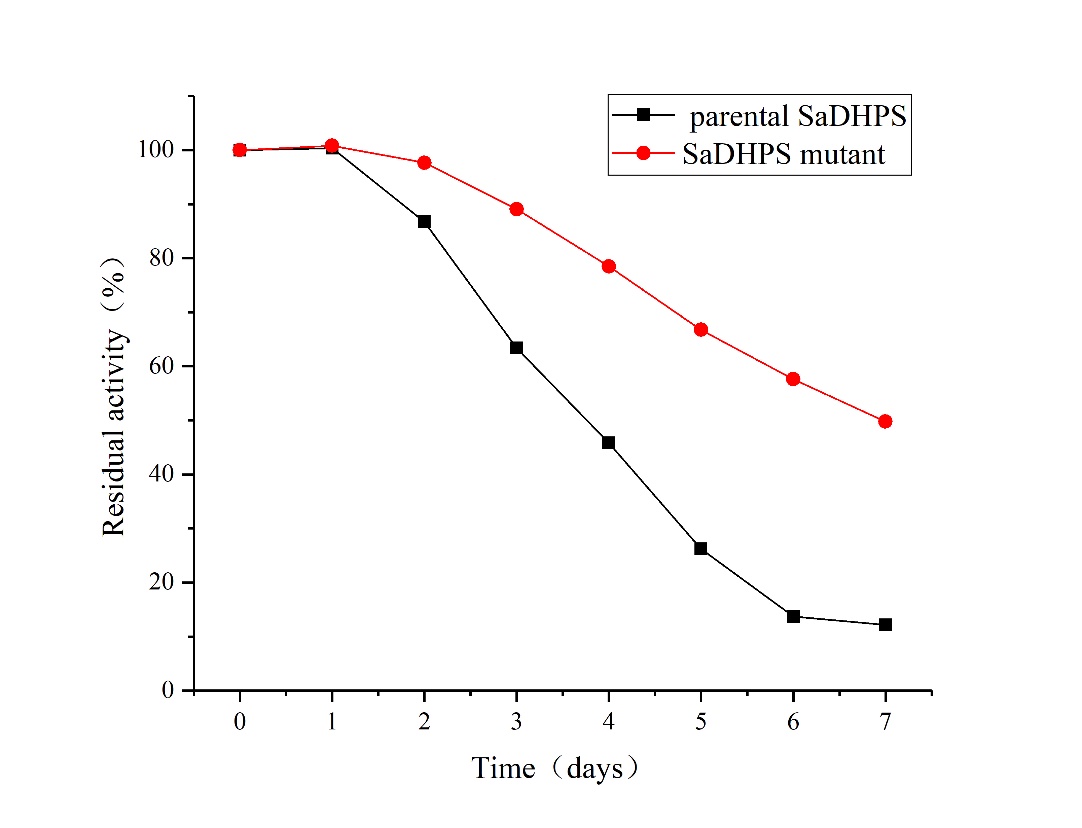


A

B






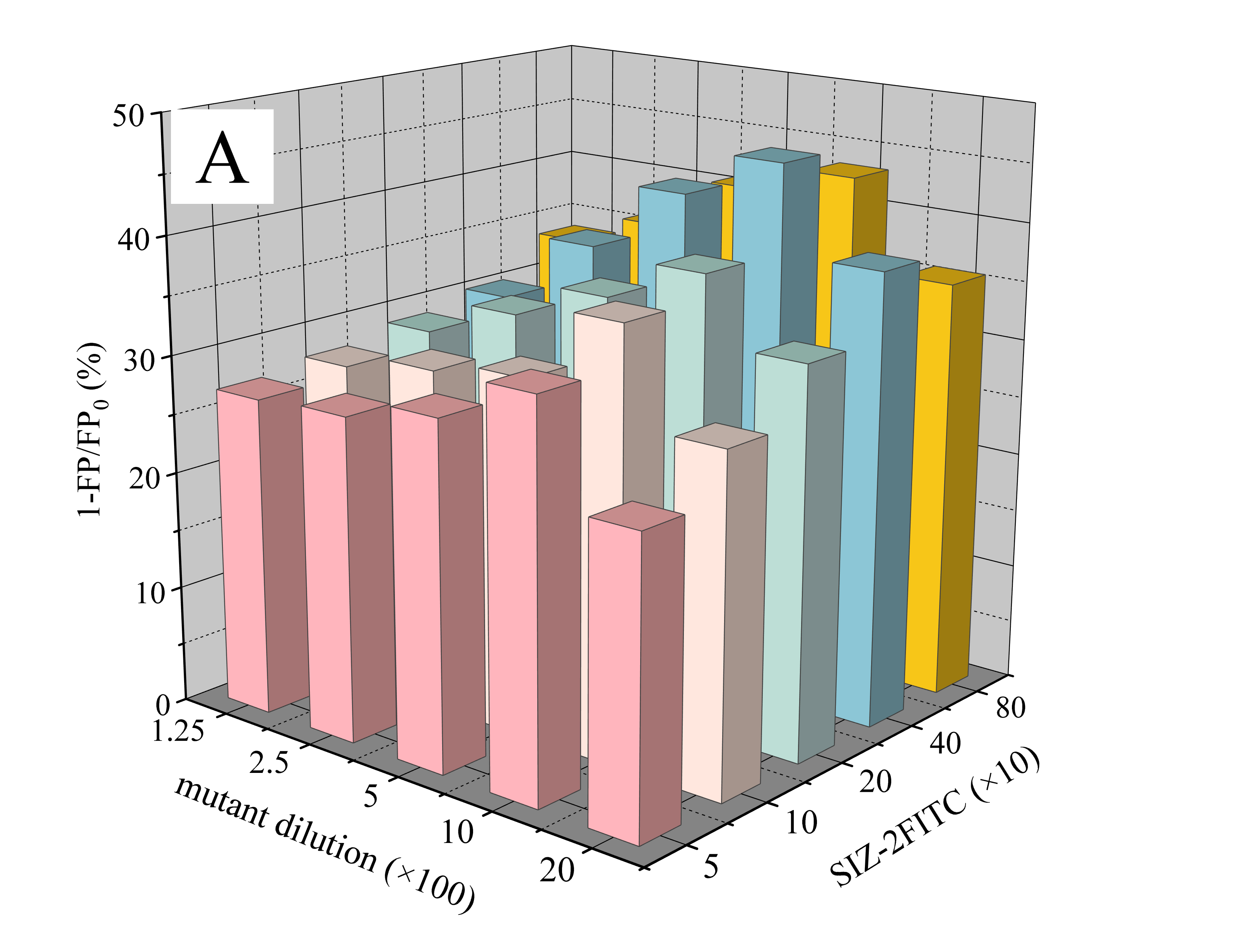


Figure S6. Results for optimization of (A) SaDHPS mutant and SIZ-2FITC (DHPPP 4 mM, MgCl_2_ 40 mM, and incubation 5min), (B) DHPPP (mutant 1:1000, SAs-2FITC 1:400, MgCl_2_ 40 mM, and incubation 5 min), (C) Mg^2+^ (mutant 1:1000, SAs-2FITC 1:400, DHPPP 4 mM, and incubation 5 min), and (D) incubation time (mutant 1:1000, SAs-2FITC 1:400, DHPPP 4 mM, and MgCl_2_ 40 mM) by using PABA (10 ng/mL).

Figure S7. Competitive inhibitory curves of SMM standard and matrix matched SMM when using the two fluorescent tracers (0.1-500 ng/mL).


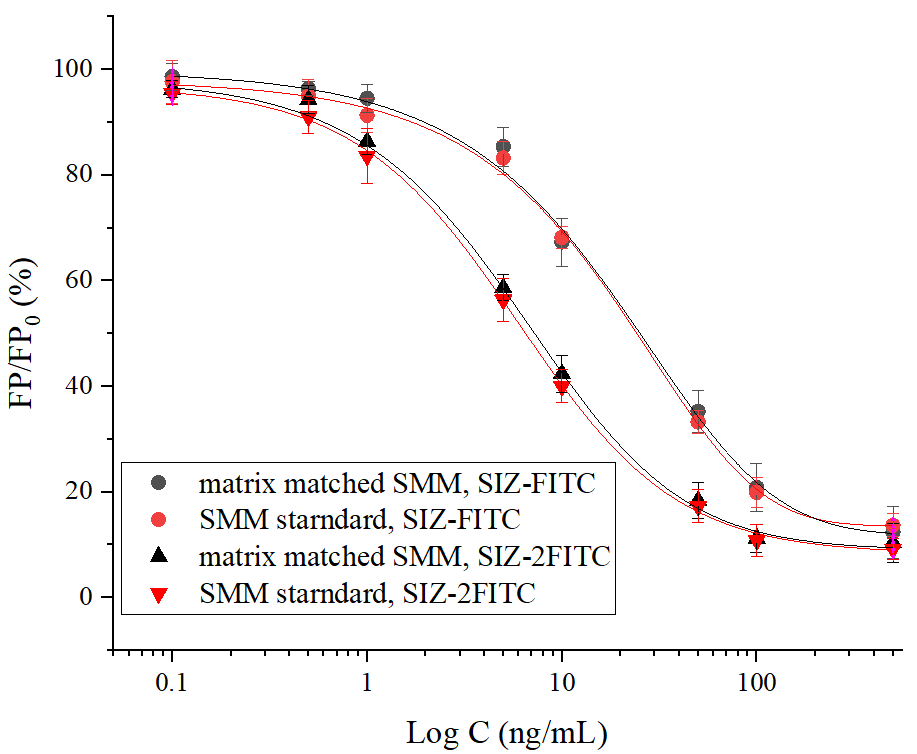


Table S1. Recoveries of the representative 8 SAs from fortified blank pork samples. (CV = coefficient of variation, n = 6)

| Analyte | Added  (ng/g) | Inter-assay | | Intra-assay | |
| --- | --- | --- | --- | --- | --- |
|  |  | Recovery  (%) | CV  (%) | Recovery  (%) | CV  (%) |
| SN | 1 | 86.5 | 9.5 | 83.2 | 9.2 |
|  | 10 | 80.2 | 8.1 | 81.0 | 17.2 |
|  | 50 | 79.5 | 13.5 | 72.2 | 11.7 |
| SA | 1 | 81.6  7 | 7.7 | 74.5 | 15.4 |
|  | 10 | 76.0 | 8.6 | 79.2 | 12.6 |
|  | 50 | 81.8 | 9.8 | 77.6 | 13.3 |
| SMZ | 1 | 83.3 | 9.2 | 88.4 | 17.7 |
|  | 10 | 77.1 | 11.2 | 82.5 | 14.5 |
|  | 50 | 86.6 | 13.8 | 81.1 | 10.7 |
| STZ | 1 | 76.2 | 10.5 | 76.2 | 9.4 |
|  | 10 | 74.4 | 11.2 | 73.3 | 7.2 |
|  | 50 | 83.5 | 16.8 | 72.5 | 7.1 |
| SD | 1 | 75.8 | 7.9 | 81.0 | 8.6 |
|  | 10 | 81.1 | 10.8 | 74.8 | 10.5 |
|  | 50 | 80.5 | 7.6 | 72.5 | 11.6 |
| SCP | 1 | 79.6 | 14.3 | 74.3 | 10.4 |
|  | 10 | 73.4 | 15.5 | 71.3 | 9.5 |
|  | 50 | 82.1 | 12.7 | 86.2 | 8.8 |
| SSZ | 1 | 77.5 | 19.6 | 77.7 | 7.1 |
|  | 10 | 76.2 | 12.5 | 80.5 | 10.2 |
|  | 50 | 83.7 | 9.0 | 75.2 | 11.8 |
| PST | 1 | 82.4 | 15.2 | 76.1 | 14.4 |
|  | 10 | 70.8 | 10.4 | 83.5 | 16.7 |
|  | 50 | 82.6 | 11.5 | 81.2 | 15.5 |

Table S2. Comparison with other FPIA and DHPS-based FPA for detection of SAs.

| Recognition reagent | method | Analyte | Assay time | LOD  (ng/g) | Ref. |
| --- | --- | --- | --- | --- | --- |
| polyclonal antibody | FPIA | 4 SAs | <10 min | 0.7-2.6 | Eremin et al., 2005 |
| polyclonal antibody | FPIA | 1 SAs | 14min | 10 | Eremin et al., 1994 |
| polyclonal antibody | FPIA | 1 SAs | --- | 0.2 | Murtazina et al., 2004 |
| monoclonal antibody | FPIA | 2 SAs | 5 min | 0.25-0.7 | Wang et al., 2007 |
| monoclonal antibody | FPIA | 3 SAs | 10 min | 0.9-3.1 | Wang et al., 2008 |
| ScFv | FPIA | 13 SAs | 5 min | 2.74-20.36 | Chen et al., 2014 |
| *S. pneumonia* DHPS | FPA | 29 SAs | 20 min | 1.6-59 | Wang et al., 2015 |
| *S. aureus* DHPS | FPA | 31 SAs | 2 min | 2.0-38.5 | He et al., 2021 |
| *S. aureus* DHPS mutant | SA-FPA | 35 SAs | 2 min | 0.03-1.16 | This study |

1. These authors contributed equally to this work. [↑](#footnote-ref-1)
2. *Corresponding author. E-mail:chinawangjp@hotmail.com (Jian Ping Wang). [↑](#footnote-ref-2)
